# Investigation of P2X7R involvement in maternal poly(i:C) exposure evoked autistic features in mice

**DOI:** 10.1186/2193-1801-4-S1-P16

**Published:** 2015-06-12

**Authors:** Gergely Horváth, Lilla Otrokocsi, Ágnes Kittel, Mária Baranyi, Beáta Sperlágh

**Affiliations:** Hungarian Academy of Sciences Institute of Experimental Medicine, Hungary

**Keywords:** Poly(I:C), Autism, P2X7R

Autism Spectrum Disorder (ASD) is a neurodevelopmental condition with core symptoms of unusual social interaction and communication and increased repetitive behaviours with limited interests of environment. Recent studies have revealed that purinergic signalling (hyperpurinergia) is one of a key features of autism. Our aim was to establish a reliable model of ASD in our lab utilizing a broad range of behavioural experiments in order to investigate the role of P2X7 in autism. We injected Poly(I:C) (PIC) in two doses to pregnant C57Bl/6 mice: 3 mg/kg on E12.5 and 1.5 mg/kg on E17.5 respectively. Offsprings were weaned 4 weeks of age and behavioural studies started from 8 weeks of age. We performed social preference test, measured the body temperature and sensorymotor coordination (rotarod). We used self grooming and marble burying test in order to investigate manifestation of repetitive behaviour and measured the sensorymotor gating with Prepulse Inhibition (PPI). After behavioural experiments animals were sacrificed. Para-sagittal sections of the cerebellar vermis were cut and Purkinje cells were counted. Synaptosome fractions were made from half brains of animals and examined by electron microscopy. Striatum and Hippocampus monoamine content were measured by HPLC. We compared PIC treated offsprings with naive animals (n=10-16 animals/group). PIC treated animals showed decreased sociability and sensorymotoric coordination but we did not find change in body temperature of PIC animals. MIA animals showed increased repetitive behaviour in the marble burying and self grooming test. Quantitative Purkinje cell dropout was found in PIC mice and electron microscopy of half brain revealed ultrastructural abnormalities in them. Higher level of monoamins were detected in ASD mice compared to the control group. Based on these results this model seem to be suitable to measure the effect of different compounds or genetic deletion on PIC induced ASD symptoms in rodents.

